# Fast, bioluminescent blinks attract group members of the nocturnal flashlight fish *Anomalops katoptron* (Bleeker, 1856)

**DOI:** 10.1186/s12983-024-00555-x

**Published:** 2025-01-13

**Authors:** Peter Jägers, Stefan Herlitze

**Affiliations:** Department of General Zoology and Neurobiology, Institute of Biology and Biotechnology, Ruhr-University Bochum, 44801 Bochum, Germany

**Keywords:** Bioluminescence, Bioluminescent signaling, Flashlight fish, *Anomalops katoptron*, Decision-making, Alarm cue

## Abstract

**Background:**

During their nighttime shoaling, the flashlight fish *Anomalops katoptron* produce fascinating, bioluminescent blink patterns, which have been related to the localization of food, determination of nearest neighbor distance, and initiation of the shoal’s movement direction. Information transfer e.g., via alarm signals is an important aspect in group living species especially when being under threat. In dark environments, bioluminescence has the potential to accurately transfer such information. Under threat *A.* *katoptron* show increased swimming speeds and a higher group cohesion accompanied by fast blink frequencies.

**Results:**

In this study we used a two-choice paradigm to test the preferences for typical blink characteristics e.g., frequency and duration. Our data show that individuals decided within short periods (< 4 s) for faster blink frequencies of artificial light organs and the preference for the higher blink frequencies became more pronounced as the difference between the presented frequencies increased. The preference correlated with the frequency rather than the duration.

**Conclusion:**

Our study suggests that fast, bioluminescent blinks of light organs lead to aggregations of *A.* *katoptron*.

**Supplementary Information:**

The online version contains supplementary material available at 10.1186/s12983-024-00555-x.

## Background

Whether under threat [1], mating [2], or foraging [3], making decisions is ubiquitous and crucial to both complex tasks and simple interactions. In schooling fish, for example, individuals must weigh the qualities of different groups that they can join [4]. To choose the most attractive group, individual needs must be balanced against aspects such as risk perception (e.g., under predation) or the availability of social information [5, 6]. In general, inter-individual differences in decision-making can be attributed to numerous factors ranging from early-life ontogenetic development [7], the ecological context [5, 8], or the architecture of neuronal circuits [9].

To reach decisions or to maintain group cohesion, intraspecific communication depending on either cues [10] and/or intentional signals [11] is necessary. Alarm signals represent an important part of intraspecific communication in birds and mammals [12], and also in fish [13]. For example, the release of chemical compounds (Schreckstoff) of fathead minnows *Pimephales promelas* [14] or visual signals such as fin-flicking in the glowlight tetra *Hemigrammus erythrozonus* [15] are used to actively warn other individuals. In context of bioluminescent signaling, a single, bright flash has been discussed as an alarm signal for the ponyfish *Gazza* *minuta* [16].

As levels of ambient light decrease, bioluminescent signals become an increasingly important source of visually guided information. In the sea, numerous protective and offensive functions of bioluminescence have been described in both non-vertebrate and vertebrate species [17]. For example, bioluminescent signals are important courtship displays [18], facilitate speciation in the deep sea [19], and allow for information exchange in social animals [20]. Furthermore, intraspecific attraction via bioluminescent flashes has been proposed for several fish species [16, 21] and is thought to have a broad implication [17]. However, precise descriptions of the interplay of e.g., pulse duration, blink frequency, or intensity that drives attraction remain scarce. In contrast to the attraction to bioluminescent light, aggressive signals can cause repulsion during territorial defense [22].

The reception of bioluminescent signals is predominantly perceived by the visual system [23] (also note [24]). The complex interaction of ambient light intensity [25], refraction through surface waves [26], and turbidity [5] can affect the perception of visual cues, thereby compromising the intentional or unintentional information provided. In dark environments, fish show several adaptations to maintain the perception of limited visual information, such as bioluminescent light. For deep-sea or nocturnal fish, these include multi-layer retinas [27], intraocular filters, which have been suggested to enhance hue discrimination [28], and expression of multiple opsins [29]. While no correlation has been found in deep-sea lanternfish (Myctophidae) [30], several species of nocturnal cardinalfish (Apogonidae) show an increased eye-to-body ratio [31]. The eye-to-body ratio in cardinalfish was primarily observed in non-bioluminescent species [31]. In the group-living, nocturnal flashlight fish *Anomalops katoptron*, opsins are tuned to visualize ambient moonlight and the emission wavelengths of their own, bioluminescent symbionts *Candidatus photodesmus katoptron* (Gammaproteobacteria: *Vibrionaceae*) [32, 33].

Hosted within the subocular light organs of *A.* *katoptron*, the symbiotic bacteria produce the continuous bioluminescent light [33, 34]. To disrupt the light emission, *A.* *katoptron* rotates its light organs to reveal the black-pigmented back of the light organ cup [35]. Multiple functions of bioluminescence occur in *A.* *katoptron*, in which alternate exposure and occlusion of light organs produce context-dependent blink patterns. These patterns have been shown to be involved in the localization of zooplankton [36], the initiation of movement direction [37], and the determination of nearest neighbor distance [38]. In the field, shoals of *A.* *katoptron* range from eight to several hundred individuals moving uncoordinated in dark caves during the day, but form highly aligned groups with increased blink frequencies when avoiding threats while schooling in the night [38]. Previous studies have focused on the functions of bioluminescent blinks when being in a group, but none have examined how bioluminescent signals drive group formation. In this paper, we used a two-choice decision-making task to investigate how bioluminescent blinks attract *A.* *katoptron*. Our results show that *A.* *katoptron* are attracted to fast blinking stimuli and that blink frequency is the most important factor.

## Methods

### Husbandry

Specimens of *Anomalops katoptron* were obtained from DeJong Marinelife (Netherlands) in April 2021, August 2022 and April 2024. Individuals were caught in the wild, and no information on age and sex was available. No sexual dimorphism was reported in previous studies [39]. Animals (*n* = 23) were maintained in smaller groups for several weeks before the experiments were carried out on the 16th, 17th, 18th and 23rd July 2021, on the 8th, 9th and 10th September 2022, and on the 17th April 2024.

The light–dark cycle was set to 12–12 h with the dark period starting at 12 h pm CET. During the day, groups of *A.* *katoptron* dwell in caves and crevices with low light intensities [38]. Therefore, we placed different shelter in the tank and installed opaque PVC cover around it. The housing tank (120 cm × 60 cm × 60 cm, L × W × H) was connected to an additional filter tank (120 cm × 60 cm × 60 cm, L × W × H). The entire system had a volume of 650 l. Standardized filter systems and aeration were used (see [36, 38] for details) to achieve steady water parameters (temperature: 25–27 °C, salinity: 34–36 ‰, NO_3_ < 20 mg/l, NO_2_ < 0.1 mg/l, PO_4_ < 0.1 mg/l). Once per dark phase, the tank was illuminated for brief intervals (< 30 s) with weak red light (TX 100; Coast; USA), and individual health was assessed. In addition, light organs were visually controlled for continuous illumination in total darkness. Twice a day, individuals were fed ad-libitum under dark conditions with defrosted zooplankton and small amounts of minced salmon. On the experiment day, animals were fed after the experiment.

### Experimental setup

**Fig. 1:**
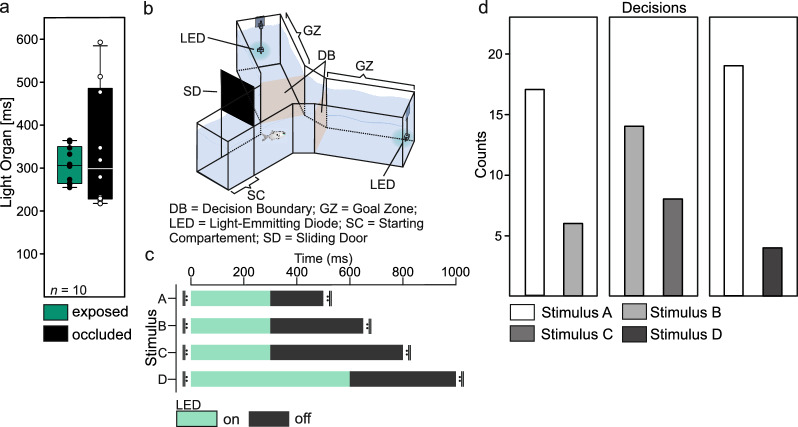
*Anomalops katoptron* show preference for fast blink frequencies. Light organ exposure and occlusion of ten individuals assigned to one of two groups was recorded prior to the experiment (**a**). A two-choice paradigm (**b**) was used to test different stimuli (**c**). Single individuals (*n* = 23) were tested for three different stimuli compositions. The total number of decisions made for each stimulus is displayed (**d**)

For our experiments, we used a radially symmetric Y-maze made from acrylic glass (wall thickness 6 mm) filled with aged water from the housing tank (water level: 15 cm). Both decision arms of the Y-maze (50 × 25 × 25 cm, L × H × W, see Fig. 1b) were equipped with an LED (Nichia 3 mm LED cyan 14.720mcd, Winger, Germany; 0.23 µW, λ_max_ = 504 nm measured in [38]), which was enclosed in an acrylic glass tube (total length 18 mm). LED intensity and wavelength were according to our previous studies, in which we performed spectrometric measurements of light organs (see [36, 38]). The inner surface of the acrylic glass tube was painted white (Email Color, Revell, Germany), to create diffuse illumination, resembling light organs of *A. katoptron*.

To control the artificial light organs, we used microcontrollers (Arduino Uno Rev3, Arduino, Italy), which were connected to a computer. We used a self-written Matlab program (Matlab 2020a, MathWorks, USA) with a graphic user interface, that allowed us to set the blink frequency, trial length, pre- and post-trial duration. To clock the LEDs, we used an additional microcontroller, which was connected via its digital outputs with the previously mentioned Arduino boards. After uploading the parameter for each LED to the Arduino boards, a pre-trial time (90 s) was started before triggering the LEDs simultaneously.

The maze was illuminated with two IR-headlights (ʎ_max_ = 850 nm; IKV ACC 07, Inkovideo GmBH, Germany) placed next to the decision-making arms on a height of 80 cm. We recorded the trials with a camera (G1X, Canon, Japan), which had the IR-filter removed, filming with a resolution of 1920 × 1280 pixel at 25 fps placed 190 cm above the maze. Additionally, we placed night shot camcorder (HDR-CX730, Sony, Japan) on a height of 15 cm next to each decision-making arm, filming the immediate blink response after the individuals decided for one side. Here, both camcorders were set to 1920 × 1280 pixel at 50 fps. Post-processing of videos (e.g., cut and convert) was carried out in Shotcut (GNU General Public License; Meltytech, LLC) or Premiere Elements 2021 (Adobe Inc., USA).

Experiments started during the dark period at 2 pm CET. To reduce effects of light pollution, the experimental area was surrounded by black sheets and all other light sources were either turned off during the experiment or covered. In addition, the computer screen was placed behind a curtain and additionally in a black, pop-up photo tent (50 × 50 × 50 cm; Walimex, Germany), which was closed during every trial. Between the trials, the photo tent was carefully opened at one side to set the new experimental conditions. This was thought to reduce the light level to a minimum.

## Experimental procedure

Prior to the two-choice experiment, the first batch of *A.* *katoptron* that arrived in our lab was randomly divided into two shoals of five individuals. To set the timing for our LED-stimuli and to compare with our previous studies, the groups were recorded for three minutes under infrared settings in a tank measuring 58 cm × 58 cm × 55 cm (L × W × H). IR-headlights and night shot camcorder were similar to the main experiment. Videos were converted in Premiere Elements 2021 to .avi files with a resolution of 1280 × 720 pixel at 25 fps.

To test the attraction for different light stimuli, each individual received three pairs of stimuli presented in a pseudorandomized order. The following combinations were presented: first combination (2 Hz: LED 300 ms on + 200 ms off vs. 1.54 Hz: LED 300 ms on + 350 ms off), second combination (1.54 Hz: LED 300 ms on + 350 ms off vs. 1.25 Hz: LED 300 ms on + 500 ms off), and third combination (2 Hz: LED 300 ms on + 200 ms off vs. 1 Hz: LED 600 ms on + 400 ms off). The orientation of the stimuli (left or right arm) was randomly switched.

Five minutes before the experiment started, individuals were transferred into the maze (Fig. 1b). After the habituation, the experiment started with a 90-s-long pre-trial time with the individual placed in the starting compartment. The LEDs were visually controlled by the experimenter and the sliding door opened immediately after the stimulus started. Each stimulus combination was analyzed for one minute. After each trial, flashlight fish were gently transferred into the starting compartment and a new pre-trial acclimatization (90 s) started. After the experiment, individuals were transferred to a separate compartment in the housing tank. This compartment (58 cm × 58 cm × 55 cm; L × W × H) was separated by an opaque PVC plate. After each individual, stock tank's water (approx. 40 l) was used to partially replace the water in the maze.

### Data analysis

For each of the ten individuals measured prior to the two-choice experiment, the light organ exposure and occlusion were noted using Solomon Coder (Version 19.08.02) and their blink frequency was calculated in Excel. Results are shown in Additional File 1 Figure S1, and raw data is included (see Additional File 2).

Lifting the door was set as the starting point and the following 60 s were analyzed. The movement trajectories were analyzed frame by frame with the video analysis software Vidana 2.0. The decision-making time was the time from leaving the refuge to approaching the goal zone. Here, the full body had to be within the goal zone. Subsequently, the time an individual spent in the goal zone was recorded. Furthermore, we recorded the blinks when the individuals decided for a stimulus (immediate blink response). Due to a short period within the goal zone no full blink was recorded in few cases (Composition 1: AK11; Composition 2: AK9 and AK10; see Additional File 2). Furthermore, the left goal zone of the maze couldn’t be recorded for individual AK23 due to a technical defect.

**Fig. 2:**
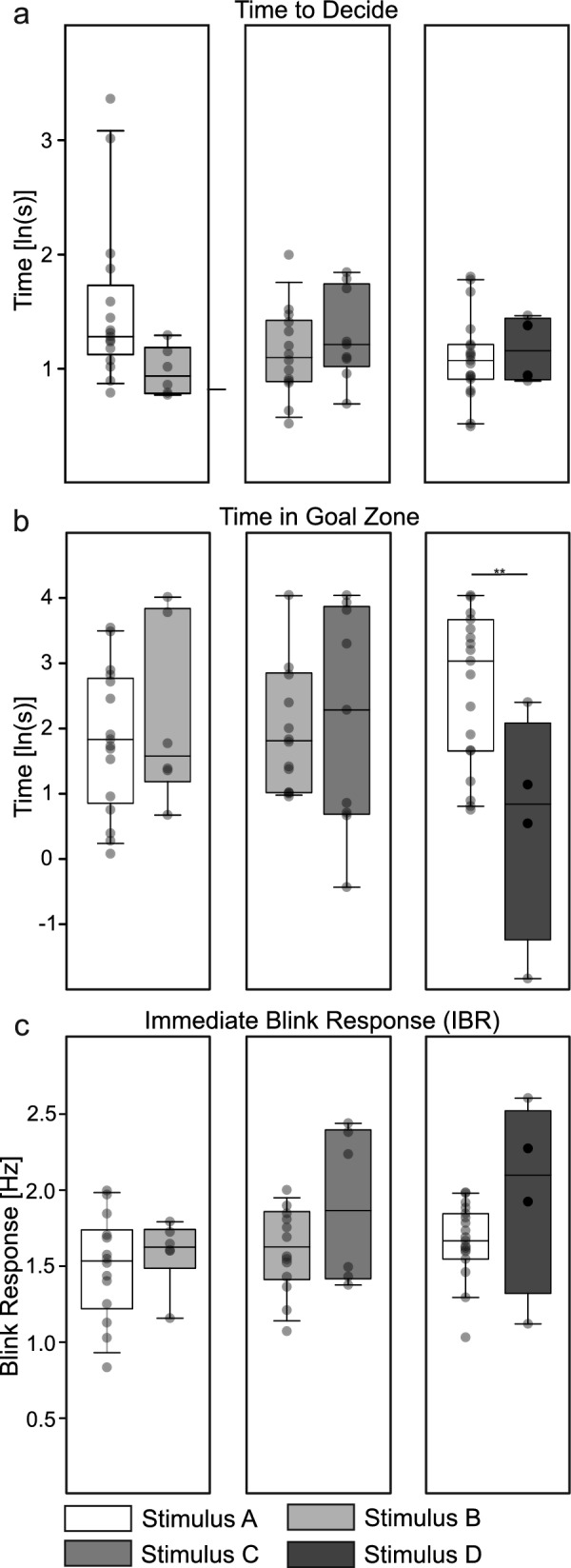
Decision time, time in goal zone, and immediate blink response (IBR) for different stimuli compositions. Each individual (*n* = 23) was tested repeatedly for three different stimulus compositions. Dots indicate data points, thereby raw data of decision-making time (**a**) and time in goal zone (**b**) were ln transformed. Individual blink frequencies were recorded from the time of entering to leaving the goal zone after the first decision regarding a stimulus was made (**c**). Significance value reported as: ** (*p* ≤ 0.01)

All statistical analyses were carried out in R version 4.3.1 [40], and results are available in the supplementary files (see Additional Files 2 and 3). The preference for a particular blink frequency (e.g., fast vs. slow) was examined using a binomial test. Here, the expected value was set to equal decisions (*p* = 0.5) for both presented stimuli. Additionally, results were adjusted using a Holm correction to reduce errors of multiple testing. The time taken to decide, time in the goal zone, and immediate blink response were analyzed using linear mixed models (LMMs) with restricted maximum likelihood in R package lme4 (version 1.1–35.3, [41]). The significance of effects was tested with Kenward-Roger F-test in package lmerTest (version 3.1–3, [42]). The model was designed with stimulus composition and decision (either fast or slow stimulus) as fixed effects, and individuals as random effect. The response variables of time to decide (Fig. 2a) and time in goal zone (Fig. 2b) were ln-transformed to improve the assumptions of normality and homogeneity of variances. For an additional pairwise comparison, we used the estimated marginal means function in package emmeans (version 1.10.1; [43]). The residuals of our models showed skewness in the histogram, indicating a predominance of small residuals and a few large ones. This skewness suggests a potential violation of the normality assumption, which could affect the accuracy of our model's predictions.

### Figures

Figures were generated in SigmaPlot (SigmaPlot 11.0; SystStat, India) and processed with CorelDraw Graphics Suite 2017 (Corel Corporation, Canada).

## Results

To investigate how the bioluminescent blinks of *A.* *katoptron* drive attraction, we used a two-choice experiment (Fig. 1b) and tested different compositions of light stimuli shown by artificial light organs (Fig. 1c). Prior to the choice experiment, we analyzed the blink frequencies which were shown by two shoals, each consisting of five individuals. The mean blink frequency was 1.62 ± 0.33 Hz (mean ± sd; see Additional File 1 Fig. S1) with light organ exposure of 307 ± 0.042 ms (mean ± sd, Fig. 1a) and occlusion of 343 ± 0.137 ms (mean ± sd, Fig. 1a). In accordance with our previous research [36, 38] and the results of the pre-experiment, we set LED on-times to 300 ms for our decision tasks (compositions 1 and 2). During intraspecific communication, a change in blink frequency is dependent on light organ occlusion [38]. Thus, we focused on different LED off-times (200, 350, 500 ms) that were adjusted to the mean $\pm$ sd of light organ occlusion shown during shoaling. Our blink frequencies were set to 1.25, 1.54 and 2 Hz. Additionally, to examine preference for a blink frequency while maintaining a similar ratio of LED on- and off-times, we adjusted the LED on-times of two stimuli to total 600 ms within a second (composition 3).

In all three decision tasks, individuals chose the fast-blinking stimulus more often (Fig. 1d). For the first stimulus composition, 17 individuals preferred the fast (2 Hz) compared to 6 individuals who chose the slow (1.54 Hz) blinking stimulus (χ^2^_1_ = 5.261, *p*_*Holm*_ = 0.044). As the difference between the presented blink frequencies decreased (second stimulus composition; 1.54 Hz vs. 1.25 Hz), the number of decisions for the fast-blinking stimulus decreased to 14 individuals (χ^2^_1_ = 1.087, *p*_*Holm*_ = 0.297). Additionally, in a composition with stimuli set to the same amount of light (600 ms out of 1000 ms) but with different frequencies (1 Hz vs. 2 Hz), individuals (*n* = 19) chose the 2 Hz stimulus more often (χ^2^_1_ = 9.783, *df* = 1, *p*_*Holm*_ = 0.005).

Next, we investigated the decision-making time after leaving the starting compartment. Across all configurations, individuals took approximately 4 s to decide (Fig. 2a). The decision-making time was slightly, but not significantly, increased when individuals chose the faster blinking stimulus within the first task.

Furthermore, we analyzed the time that individuals spent in the chosen target zone. No differences were found for the first and second task. For the third composition, individuals spent more time in the target zone when they chose the 2 Hz stimulation (Fig. 2b; emmeans: t(52) = −4.12, *p* = 0.002). Finally, we analyzed the rate of bioluminescent blinks of the individuals immediately after making their decision. Summarizing all responses, individuals showed a blinking rate of 1.67 ± 0.42 Hz (mean ± sd). This was comparable to the group recordings prior to the two-choice experiment and didn’t differ significantly within (Fig. 2c; composition 1: t(58.5) = −0.409, *p* = 1.00; composition 2: t(58.4) = 1.31, *p* = 0.96; composition 3: t(58.7) = 1.55, *p* = 0.87) and between the stimulus compositions. Although the difference did not reach statistical significance, there appeared to be a tendency for individuals to choose for the slow-flashing stimulus to have a higher blink frequency than individuals choosing the fast-flashing stimulus.

## Discussion

To maximize the benefits of living in a group, animals rely on intraspecific communication either through cues or signals [44, 45]. In dark environments, bioluminescence has the potential to accurately convey visual information [17]. Although most studies on the ability to communicate via bioluminescence have been conducted in terrestrial insects (Coleoptera: Lampyridae) [46], marine organisms also show bioluminescent signaling [17]. For example, polychaetes, crustaceans, and echinoderms have been reported to transmit information via bioluminescence [17]. In addition, a complex system of information transfer combining pigmentation patterns and light emission has been described in squid [20]. Several fish species use timed flashes that allow to maintain aggregation (e.g., Leiognathidae) [16] or to recognize conspecifics (e.g., Myctophidae) [21]. Of course, for most species it hasn’t been investigated whether these flashes are passive cues or active signals. In the flashlight fish *Anomalops katoptron* the use of an active signaling mechanism is most likely because bioluminescent blinks are context-dependent, can be actively regulated, and can influence the behavior of the receiver [38].

In this paper we show that individual *A.* *katoptron* were attracted to fast pulses of artificial light organs in a two-choice task and most decisions were made in less than four seconds. In general, individuals made decisions in all trials indicating a high motivation to aggregate with artificial conspecifics. Although tested in small shoals, individuals in the lab exhibit lower blink frequencies compared to larger populations in the field [38]. The stimuli used in our study were adjusted to match tank conditions. A blink frequency of 2 Hz, as presented in this study, is observed in naturally occurring groups while shoaling in caves during the day. Furthermore, it falls within the lower quartile of frequencies observed in groups shoaling on reef flats in the Indo-Pacific at night [38].

In our experiment, the choice for the faster blinking LEDs correlated with the frequency difference between the presented stimuli. It was strongest for composition three (difference: 1 Hz) and weakest for composition two (difference: 0.25 Hz). Signal discrimination is related to physiological limitations of the sensory system e.g., stimuli don’t reach the threshold required to elicit a behavioral response [47]. In *A.* *katoptron*, electrophysiological and additional HEK cell recordings suggest that opsins are tuned to blue light (~ 490 nm) and short pulses of > 10 ms can evoke noticeable currents [32]. How the visual system shapes the decision-making process in *A.* *katoptron* remains speculative at this point, but the ability to discriminate small differences in light signals is very likely. Light organs of starved individuals showed decreased luminescence [48]. It will be interesting to investigate the influence of light organ intensity on the decision-making process.

In situ experiments showed that individuals of larger groups of *A.* *katoptron* increased their blink frequencies and formed denser aggregations during escape responses [37, 38]. Similarly, increased blink frequencies were observed in the tank when reciprocal interaction with conspecifics was not allowed e.g., when being isolated. In addition, nearest neighbor distance was negatively correlated with blink frequency [38]. Closer orientation to conspecifics has been observed in many species, either as a long-term response in high-predation habitats [49] or as a short-term response to threat [50], and is considered to be an adaptive mechanism to stress. In *A.* *katoptron*, it has therefore been proposed that fast blink frequencies are associated with stress [37, 38].

Increased blink frequencies emitted by an individual may indicate a selfish signal, e.g., to recruit more individuals, thus benefiting the emitter from group related factors, or act as an alarm signal warning other individuals [13]. Alarm signals in fish have been frequently associated with dense shoaling. For example, when glowlight tetra *Hemigrammus erythrozonus* detected fin-flicking, an alarm cue presented after exposure to predator odors, they exhibited freezing behaviors and shoaled more cohesively [15]. Similarly, alarm cues presented to the x-ray tetra *Pristella maxillaris* resulted in denser schools and individuals increased probability of being observed by neighbors [51]. In context of bioluminescent signaling, a single flash of the ponyfish *Gazza* *minuta* has previously been interpreted as an alarm signal but has also been discussed as a startle response [16]. Atlantic midshipman *Porichthys porosissimus* emit bioluminescent flashes when attacked by predators, which Lane (1967) discussed as an aposematic signal to advertise the toxic opercular spine [52, 53]. Smith (1992) mentioned that these flashes have the potential to be an alarm signal or serve as a distress call [13]. Whether the increased blink frequency of *A.* *katoptron* is also directed at predators, i.e., to show that individuals are alerted, and how this affects the predator–prey interaction will be an exciting field for future studies. Individuals of *A.* *katoptron* preferably shoal with conspecifics that exhibit increased blink frequencies in response to threats. This behavior indicates that the receiving individuals are responding to the increased blink frequencies, suggesting that this system could function as an alarm signaling mechanism.

In our experiment, the immediate blink response was nearly consistent across trials and similar to the frequencies measured a priori or in previous studies [38]. Since the blink frequency of the individual did not change when attracted by high-frequency blinking, an important question arises: would this behavior reduce the overall quality of the group and ultimately decrease the individual ‘s fitness? Differences in frequency between the individual and the light organ dummy indicate that individuals did not show copying behavior with respect to blink frequency, unlike schools of *A.* *katoptron* that synchronize during escape responses [37], other species that use bioluminescent signals for communication such as male ostracods during mating [54], or non-marine organisms such as fireflies [46]. Our results suggest that individuals do not promote information from others, and that other cues may need to coexist to induce a rapid blink frequency. Previous studies showed that information from other individuals needs to match the individual’s perception, for example, of a predator, to elicit an anti-predator response [55]. Asynchronous blinking generates visual clutter, potentially reducing the accuracy of information transfer (e.g., as observed in fireflies *Photinus* *carolinus* [56]) and might decrease the benefits of living in a group. Furthermore, asynchronous blinking, coupled with changes in swimming direction- as seen in *A.* *katoptron* [57]- holds the potential to confuse visual predators. The benefits of synchronized versus noisy blinking in groups of flashlight fish under ecologically relevant conditions should be investigated in future studies.

In general, joining a group provides benefits such as the safety in numbers or the confusion effect [58]. In addition, larger, more cohesive groups can gather information faster and decisions can be more accurate i.e., through consensus decision-making [59]. In our study, the attraction to fast blinks may also be related to the closer orientation between individuals that these signals promote. In freely moving *A.* *katoptron*, fast blink frequencies are associated with escape responses [37, 38]. Therefore, fast frequencies might indicate the departure of a distancing group, which in turn triggers other group members to change their swimming direction accordingly [37].

Although we observed clear preferences for the decision itself, we did not see differences in other important parameters of decision-making task, i.e., decision-making time. Improved sample size could give a more detailed perspective. In our study, *A.* *katoptron* were only occasionally available, were wild-caught and arrived in several batches throughout multiple years in our laboratory. For this reason, it would be interesting to test individuals sharing a similar and/or controlled life-history. Additionally, it would be interesting to compare these results between different shoals to test if each shoal establishes its own fundamental blink frequency, shifting the preference towards a particular blink frequency. Furthermore, it would be interesting to establish dynamic light-organ responses to analyze reciprocal interactions following a decision. Our setup was set to fixed stimuli that might have caused familiarization or repulsion due to its static frequency. We tested only a subset of possible blink compositions. Future studies exploring a broader range of blink frequencies should be carefully designed, utilizing fully factorial designs and repeated measurements.

## Conclusions

Bioluminescence is a common feature among marine fish, yet little is known about the bioluminescent signaling mechanisms that underlie group formation. Our findings reveal that fast bioluminescent blinks play an important role in the aggregation of flashlight fish *A.* *katoptron*. While we observed significant differences in their choice related to blink frequencies, other behavioral traits i.e. time to make a choice were not significant. Despite the limitation of a small sample size, these results contribute to the understanding of how specific bioluminescent flashes can facilitate group formation in visually restricted habitats. Moreover, they will help to establish experimental paradigms for other bioluminescent species such as ponyfish (Leiognathidae) or lanternfish (Myctophidae).

## Supplementary Information

Additional file 1.

Additional file 2.

Additional file 3.

## Data Availability

The datasets supporting the conclusions of this article are included within the article (and its additional files).

## References

[1] Hein AM, Gil MA, Twomey CR, Couzin ID, Levin SA. Conserved behavioral circuits govern high-speed decision-making in wild fish shoals. Proc Natl Acad Sci. 2018;115:12224–8. 10.1073/pnas.1809140115. 30420510 10.1073/pnas.1809140115 PMC6275531

[2] Amundsen T, Forsgren E. Male mate choice selects for female coloration in a fish. Proc Natl Acad Sci. 2001;98:13155–60. 10.1073/pnas.211439298. 11606720 10.1073/pnas.211439298 PMC60840

[3] Ward AJW, Krause J, Sumpter DJT. Quorum decision-making in foraging fish shoals. PLoS ONE. 2012;7: e32411. 10.1371/journal.pone.0032411. 22412869 10.1371/journal.pone.0032411 PMC3296701

[4] Krause J, Rubenstein D, Brown D. Shoal choice behaviour in fish: The relationship between assessment time and assessment quality. Behaviour. 1997;134:1051–62. 10.1163/156853997X00395.

[5] Chamberlain AC, Ioannou CC. Turbidity increases risk perception but constrains collective behaviour during foraging by fish shoals. Animal Behav. 2019. 10.1016/j.anbehav.2019.08.012.

[6] Dall SRX, Giraldeau L-A, Olsson O, McNamara JM, Stephens DW. Information and its use by animals in evolutionary ecology. Trends Ecol Evol. 2005;20:187–93. 10.1016/j.tree.2005.01.010. 16701367 10.1016/j.tree.2005.01.010

[7] Laskowski KL, Bierbach D, Jolles JW, Doran C, Wolf M. The emergence and development of behavioral individuality in clonal fish. Nat Commun. 2022;13:6419. 10.1038/s41467-022-34113-y. 36307437 10.1038/s41467-022-34113-y PMC9616841

[8] Herbert-Read JE, Wade ASI, Ramnarine IW, Ioannou CC. Collective decision-making appears more egalitarian in populations where group fission costs are higher. Biol Let. 2019;15:20190556. 10.1098/rsbl.2019.0556. 31847746 10.1098/rsbl.2019.0556 PMC6936019

[9] Fernandes AM, Mearns DS, Donovan JC, Larsch J, Helmbrecht TO, Kölsch Y, et al. Neural circuitry for stimulus selection in the zebrafish visual system. Neuron. 2021;109:805-822.e6. 10.1016/j.neuron.2020.12.002. 33357384 10.1016/j.neuron.2020.12.002

[10] Lemasson B, Tanner C, Woodley C, Threadgill T, Qarqish S, Smith D. Motion cues tune social influence in shoaling fish. Sci Rep. 2018;8:9785. 10.1038/s41598-018-27807-1. 29955069 10.1038/s41598-018-27807-1 PMC6023868

[11] Vail AL, Manica A, Bshary R. Referential gestures in fish collaborative hunting. Nat Commun. 2013;4:1765. 10.1038/ncomms2781. 23612306 10.1038/ncomms2781

[12] Zuberbühler K. Chapter 8 survivor signals: the biology and psychology of animal alarm calling. In: Advances in the study of behavior. Cambridge: Academic Press; 2009. p. 277–322.

[13] Smith RJF. Alarm signals in fishes. Rev Fish Biol Fisheries. 1992;2:33–63. 10.1007/BF00042916.

[14] Bairos-Novak KR, Ferrari MCO, Chivers DP. A novel alarm signal in aquatic prey: familiar minnows coordinate group defences against predators through chemical disturbance cues. J Anim Ecol. 2019;88:1281–90. 10.1111/1365-2656.12986. 30997683 10.1111/1365-2656.12986

[15] Brown GE, Godin J-GJ, Pedersen J. Fin-flicking behaviour: a visual antipredator alarm signal in a characin fish *Hemigrammus**erythrozonus*. Animal Behav. 1999;58:469–75. 10.1006/anbe.1999.1173. 10.1006/anbe.1999.1173 10479362

[16] McFall-Ngai MJ, Dunlap PV. Three new modes of luminescence in the leiognathid fish *Gazza minuta*: discrete projected luminescence, ventral body flash, and buccal luminescence. Mar Biol. 1983;73:227–37. 10.1007/BF00392247.

[17] Haddock SHD, Moline MA, Case JF. Bioluminescence in the sea. Ann Rev Mar Sci. 2010;2:443–93. 10.1146/annurev-marine-120308-081028. 21141672 10.1146/annurev-marine-120308-081028

[18] Ellis EA, Oakley TH. High rates of species accumulation in animals with bioluminescent courtship displays. Curr Biol. 2016;26:1916–21. 10.1016/j.cub.2016.05.043. 27345160 10.1016/j.cub.2016.05.043

[19] Davis MP, Holcroft NI, Wiley EO, Sparks JS, Leo SW. Species-specific bioluminescence facilitates speciation in the deep sea. Mar Biol. 2014;161:1139–48. 10.1007/s00227-014-2406-x. 24771948 10.1007/s00227-014-2406-x PMC3996283

[20] Burford BP, Robison BH. Bioluminescent backlighting illuminates the complex visual signals of a social squid in the deep sea. Proc Natl Acad Sci. 2020;117:8524–31. 10.1073/pnas.1920875117. 32205436 10.1073/pnas.1920875117 PMC7165453

[21] Mensinger AF, Case JF. Luminescent properties of deep sea fish. J Exp Mar Biol Ecol. 1990;144:1–15. 10.1016/0022-0981(90)90015-5.

[22] Hellinger J, Jägers P, Spoida K, Weiss LC, Mark MD, Herlitze S. Analysis of the territorial aggressive behavior of the bioluminescent flashlight fish *Photoblepharon steinitzi* in the red sea. Front Mar Sci. 2020;7:78. 10.3389/fmars.2020.00078.

[23] Meyer-Rochow V, Baburina V, Smirnov S. Histological observations on the eyes of the two luminescent fishes *Photoblepharon palpebratus* (Boddaert) and *Anomalops katoptron* (Blkr.). Zoologischer Anzeiger. A Journal of Comparative Zoology. 209 (1982).

[24] Meyer-Rochow V. Fish chromatophores as sensors of environmental stimuli. In: Kapoor BG, Hara TJ, editors. Sensory Biology of jawed fishes. Enfield NH: Science Publ; 2001. p. 317–34.

[25] Ryer CH, Olla BL. Effect of light on juvenile walleye pollock shoaling and their interaction with predators. Mar Ecol Prog Ser. 1998;167:215–26. 10.3354/meps167215.

[26] Attwell JR, Ioannou CC, Reid CR, Herbert-Read JE. Fish avoid visually noisy environments where prey targeting is reduced. Am Nat. 2021;198:421–32. 10.1086/715434. 34403312 10.1086/715434

[27] Warrant EJ, Locket NA. Vision in the deep sea. Biol Rev Camb Philos Soc. 2004;79:671–712. 10.1017/s1464793103006420. 15366767 10.1017/s1464793103006420

[28] de Busserolles F, Fogg L, Cortesi F, Marshall J. The exceptional diversity of visual adaptations in deep-sea teleost fishes. Semin Cell Dev Biol. 2020;106:20–30. 10.1016/j.semcdb.2020.05.027. 32536437 10.1016/j.semcdb.2020.05.027

[29] Musilova Z, Cortesi F, Matschiner M, Davies WIL, Patel JS, Stieb SM, et al. Vision using multiple distinct rod opsins in deep-sea fishes. Science. 2019;364:588–92. 10.1126/science.aav4632. 31073066 10.1126/science.aav4632 PMC6628886

[30] de Busserolles F, Fitzpatrick JL, Paxton JR, Marshall NJ, Collin SP. Eye-size variability in deep-sea lanternfishes (*Myctophidae*): an ecological and phylogenetic study. PLoS ONE. 2013;8: e58519. 10.1371/journal.pone.0058519. 23472203 10.1371/journal.pone.0058519 PMC3589346

[31] Fishelson L, Ayalon G, Zverdling A, Holzman R. Comparative morphology of the eye (with particular attention to the retina) in various species of cardinal fish (*Apogonidae*, *Teleostei*). Anat Rec A Discov Mol Cell Evol Biol. 2004;277:249–61. 10.1002/ar.a.20005. 15052652 10.1002/ar.a.20005

[32] Mark MD, Donner M, Eickelbeck D, Stepien J, Nowrousian M, Kück U, et al. Visual tuning in the flashlight fish *Anomalops katoptron* to detect blue, bioluminescent light. PLoS ONE. 2018;13: e0198765. 10.1371/journal.pone.0198765. 29995896 10.1371/journal.pone.0198765 PMC6040694

[33] Haneda Y, Tsuji FI. Light production in the luminous fishes Photoblepharon and Anomalops from the Banda Islands. Science. 1971;173:143–5. 10.1126/science.173.3992.143. 5581906 10.1126/science.173.3992.143

[34] Hendry TA, de Wet JR, Dougan KE, Dunlap PV. Genome evolution in the obligate but environmentally active luminous symbionts of flashlight fish. Genome Biol Evol. 2016;8:2203–13. 10.1093/gbe/evw161. 27389687 10.1093/gbe/evw161 PMC4987116

[35] Johnson GD, Rosenblatt RH. Mechanisms of light organ occlusion in flashlight fishes, family Anomalopidae (*Teleostei*: *Beryciformes*), and the evolution of the group. Zool J Linn Soc. 1988;94:65–96. 10.1111/j.1096-3642.1988.tb00882.x.

[36] Hellinger J, Jägers P, Donner M, Sutt F, Mark MD, Senen B, et al. The flashlight fish *Anomalops katoptron* uses bioluminescent light to detect prey in the dark. PLoS ONE. 2017;12: e0170489. 10.1371/journal.pone.0170489. 28178297 10.1371/journal.pone.0170489 PMC5298212

[37] Gruber DF, Phillips BT, O’Brien R, Boominathan V, Veeraraghavan A, Vasan G, et al. Bioluminescent flashes drive nighttime schooling behavior and synchronized swimming dynamics in flashlight fish. PLoS ONE. 2019;14: e0219852. 10.1371/journal.pone.0219852. 31412054 10.1371/journal.pone.0219852 PMC6693688

[38] Jägers P, Wagner L, Schütz R, Mucke M, Senen B, Limmon V, et al. Social signaling via bioluminescent blinks determines nearest neighbor distance in schools of flashlight fish *Anomalops katoptron*. Sci Rep. 2021;11:1–12. 10.1038/s41598-021-85770-w. 33742043 10.1038/s41598-021-85770-w PMC7979757

[39] Steche O. Die Leuchtorgane von *Anomalops katoptron* und *Photoblepharon palpebratus*, zwei Oberflächenfischen aus dem Malaiischen Archipel. Ein Beitrag zur Morphologie und Physiologie der Leuchtorgane der Fische. Zeitschrift für wissenschaftliche Zoologie. 1909:349–408.

[40] R Core Team. R: a language and environment for statistical computing. Vienna, Austria: R Foundation for Statistical Computing (2023).

[41] Bates D, Mächler M, Bolker B, Walker S. Fitting linear mixed-effects models using lme4. J Stat Soft. 2015. 10.18637/jss.v067.i01.

[42] Kuznetsova A, Brockhoff PB, Christensen RHB. lmerTest package: tests in linear mixed effects models. J Stat Soft. 2017. 10.18637/jss.v082.i13.

[43] Russell V. Lenth. Estimated marginal means, aka least-squares means [R package emmeans version 1.10.1]: comprehensive R archive network (CRAN); (2024).

[44] Siebeck UE, Parker AN, Sprenger D, Mäthger LM, Wallis G. A species of reef fish that uses ultraviolet patterns for covert face recognition. Curr Biol. 2010;20:407–10. 10.1016/j.cub.2009.12.047. 20188557 10.1016/j.cub.2009.12.047

[45] Bachmann JC, Cortesi F, Hall MD, Marshall NJ, Salzburger W, Gante HF. Real-time social selection maintains honesty of a dynamic visual signal in cooperative fish. Evol Lett. 2017;1:269–78. 10.1002/evl3.24. 30283655 10.1002/evl3.24 PMC6121853

[46] Lewis SM, Cratsley CK. Flash signal evolution, mate choice, and predation in fireflies. Annu Rev Entomol. 2007;53:293–321. 10.1146/annurev.ento.53.103106.093346. 10.1146/annurev.ento.53.103106.093346 17877452

[47] Kasumyan AO. The olfactory system in fish: structure, function, and role in behavior. J Ichthyol. 2004;44:S180.

[48] Meyer-Rochow VB. Loss of bioluminescence in Anomalops katoptron due to starvation. Experientia. 1976;32:1175–6. 10.1007/BF01927610.

[49] Herbert-Read JE, Rosén E, Szorkovszky A, Ioannou CC, Rogell B, Perna A, et al. How predation shapes the social interaction rules of shoaling fish. Proc R Soc B. 2017;284:20171126. 10.1098/rspb.2017.1126. 28855361 10.1098/rspb.2017.1126 PMC5577484

[50] Romenskyy M, Herbert-Read JE, Ioannou CC, Szorkovszky A, Ward AJW, Sumpter DJT. Quantifying the structure and dynamics of fish shoals under predation threat in three dimensions. Behav Ecol. 2020;31:311–21. 10.1093/beheco/arz197.

[51] Schaerf TM, Dillingham PW, Ward AJW. The effects of external cues on individual and collective behavior of shoaling fish. Sci Adv. 2017;3: e1603201. 10.1126/sciadv.1603201. 28691088 10.1126/sciadv.1603201 PMC5482554

[52] Lane ED. A study of the Atlantic midshipmen, *Porichthys porosissimus*, in the vicinity of Port Aransas, Texas. Texas Contrib. Mar. Sci. 1967:1–53.

[53] Cormier MJ, Crane JM, Nakano Y. Evidence for the identity of the luminescent systems of Porichthys porosissimus (fish) and Cypridina hilgendorfii (*crustacean*). Biochem Biophys Res Commun. 1967;29:747–52. 10.1016/0006-291X(67)90281-1. 5624784 10.1016/0006-291x(67)90281-1

[54] Hensley NM, Rivers TJ, Gerrish GA, Saha R, Oakley TH. Collective synchrony of mating signals modulated by ecological cues and social signals in bioluminescent sea fireflies. Proc R Soc B. 2023;290:20232311. 10.1098/rspb.2023.2311. 38018106 10.1098/rspb.2023.2311 PMC10685132

[55] Magurran AE, Higham A. Information transfer across fish shoals under predator threat. Ethology. 1988;78:153–8. 10.1111/j.1439-0310.1988.tb00226.x.

[56] Moiseff A, Copeland J. Firefly synchrony: a behavioral strategy to minimize visual clutter. Science. 2010;329:181. 10.1126/science.1190421. 20616271 10.1126/science.1190421

[57] Jägers P, Frischmuth T, Herlitze S. Correlation between bioluminescent blinks and swimming behavior in the splitfin flashlight fish Anomalops katoptron. BMC Ecol Evol. 2024;24:97. 10.1186/s12862-024-02283-6. 38987674 10.1186/s12862-024-02283-6 PMC11234731

[58] Krause J, Ruxton GD. Living in groups. 1st ed. Oxford: Oxford Univ. Press; 2002.

[59] Sumpter DJT, Krause J, James R, Couzin ID, Ward AJW. Consensus decision making by fish. Curr Biol. 2008;18:1773–7. 10.1016/j.cub.2008.09.064. 19013067 10.1016/j.cub.2008.09.064

