# Supplementary material for: Fast, bioluminescent blinks attract group members of the nocturnal flashlight fish Anomalops katoptron (Bleeker, 1856)
Source: Front Zool. 2025 Jan 13;22:1. doi: 10.1186/s12983-024-00555-x (PMC11727482; doi:10.1186/s12983-024-00555-x)
Supplement: Supplementary file 1 — Additional file 1. [file 12983_2024_555_MOESM1_ESM.pdf]

Additional File 1 for the manuscript:

Fast, bioluminescent blinks attract group members of the nocturnal flashlight fish *Anomalops katoptron* (Bleeker, 1856)

Peter Jägers [1] ** & Stefan Herlitze [1] *

[1] Department of General Zoology and Neurobiology, Institute of Biology and Biotechnology, Ruhr-University Bochum, 44801 Bochum, Germany

* Correspondence: sxh106@gmail.com

** Correspondence: peter.jaegers@ruhr-uni-bochum.de

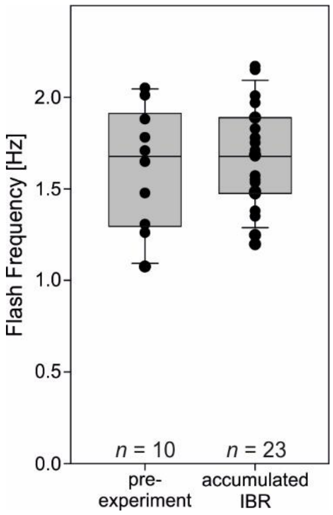

Figure S1: Blink frequencies of *A. katoptron* prior to and during the experiment. Shown are the blink frequencies of ten individuals measured in two groups prior to the two-choice experiment. Individuals were only occasionally available at the distributor and arrived in different batches. Therefore, we determined the light organ exposure/occlusion of the first ten individuals (see main manuscript). Additionally, we summarized the individual's blink frequency (*n* = 23) after deciding for a goal zone in our experiment. Results show that no difference in flash frequency occurs between being in a group before the experiment and the IBR (immediate blink response) in the two-choice experiment.
