# Supplementary material for: Fast, bioluminescent blinks attract group members of the nocturnal flashlight fish Anomalops katoptron (Bleeker, 1856)
Source: Front Zool. 2025 Jan 13;22:1. doi: 10.1186/s12983-024-00555-x (PMC11727482; doi:10.1186/s12983-024-00555-x)
Supplement: Supplementary file 3 — Additional file 3. [file 12983_2024_555_MOESM3_ESM.pdf]

Additional File 3 for the manuscript:

Fast, bioluminescent blinks attract group members of the nocturnal flashlight fish *Anomalops katoptron* (Bleeker, 1856)

Peter Jägers [1]** & Stefan Herlitze [1]*

[1] Department of General Zoology and Neurobiology, Institute of Biology and Biotechnology, Ruhr-University Bochum, 44801 Bochum, Germany

* Correspondence: sxh106@gmail.com

** Correspondence: peter.jaegers@ruhr-uni-bochum.de

This document contains the code required for the statistical analysis of our raw data (Additional File2.xlsx) in RStudio (version 2024.04.0+735, 'Chocolate Cosmos' release; R Core Team, Austria).

```
##########################################################################################

## Manuscript: Fast, bioluminescent flashes attract group members of the nocturnal flashlight fish
```

Anomalops katoptron (Bleeker, 1856)

```
## Authors: Peter Jägers and Stefan Herlitze

## Analysis Author: P. Jägers

## Analysis Start Date: April 2024

## RStudio 2024.04.0+735 "Chocolate Cosmos" Release

##########################################################################################

rm(list=ls())

library(readxl)

library(lme4)

library(lmerTest)

library(emmeans)

##########################################################################################
```

Statistical Analysis for Figure 2d

```
##########################################################################################

comb1 <- prop.test(17, 23, p = NULL, alternative = c("two.sided"), conf.level =0.95, correct = FALSE)

comb2 <- prop.test(14, 23, p = NULL, alternative = c("two.sided"), conf.level =0.95, correct = FALSE)
```

```
comb3 <- prop.test(19, 23, p = NULL, alternative = c("two.sided"), conf.level =0.95, correct = FALSE)

p_values <- c(comb1$p.value, comb2$p.value, comb3$p.value)

p.adjust(p_values, method = "holm")
```

##

Statistical Analysis for Figure 3a

##

```
Time_To_Decide_new <- read_excel("Additional File 2.xlsx", sheet = "TimeToDecide")

lat <- as.data.frame(Time_To_Decide_new)

lat$Stimulus <- as.factor(lat$Stimulus)

lat$Decision <- as.factor(lat$Decision)

latency <- lmer(LnTrans ~  Decision * Stimulus + (1|Subject), data = lat)

summary(latency)

anova(latency, ddf = "Kenward-Roger")

em1 <- emmeans(latency, c("Decision", "Stimulus"))

contrast(em1)

contrast(em1, method = "pairwise", adjust = "Sidak")

hist(resid(latency))
```

```
##########################################################################################
Statistical Analysis for Figure 3b
##########################################################################################

Time_in_Box <- read_excel("Additional File 2.xlsx", sheet ="TimeInBox")

stay <- as.data.frame(Time_in_Box)

stay$Stimulus <- as.factor(stay$Stimulus)

stay$Decision <- as.factor(stay$Decision)

stay_in_goalzone <- lmer(LnTrans2 ~  Decision * Stimulus + (1|Subject), data = stay)

summary(stay_in_goalzone)

anova(stay_in_goalzone, ddf = "Kenward-Roger")

em2 <- emmeans(stay_in_goalzone, c("Decision", "Stimulus"))

contrast(em2)

contrast(em2, method = "pairwise", adjust = "Sidak")

hist(resid(stay_in_goalzone))

##########################################################################################
Statistical Analysis for Figure 3c
##########################################################################################

blink_frequency <- read_excel("Additional File 2.xlsx", sheet ="IBR")

blink <- as.data.frame(blink_frequency)

blink$Stimulus <- as.factor(blink$Stimulus)
```

```
blink$Decision <- as.factor(blink$Decision)

IFR <- lmer(IBR ~  Decision * Stimulus + (1|Subject), data = blink)

summary(IFR)

anova(IFR, ddf = "Kenward-Roger")

em3 <- emmeans(IFR, c("Decision", "Stimulus"))

contrast(em3)

contrast(em3, method = "pairwise", adjust = "Sidak")

hist(resid(IFR))
```
